# Sodium–Glucose CoTransporter-2 Inhibitor Empagliflozin Ameliorates Sunitinib-Induced Cardiac Dysfunction via Regulation of AMPK–mTOR Signaling Pathway–Mediated Autophagy

**DOI:** 10.3389/fphar.2021.664181

**Published:** 2021-04-29

**Authors:** Changzhen Ren, Kaiqiang Sun, Yanda Zhang, Yangxi Hu, Bowen Hu, Jian Zhao, Zhiqing He, Ru Ding, Weizhong Wang, Chun Liang

**Affiliations:** ^1^ Department of Cardiology, Changzheng Hospital, Naval Medical University, Shanghai, China; ^2^ Department of General Practice, 960th Hospital of PLA, Jinan, China; ^3^ Department of Spine Surgery, Changzheng Hospital, Naval Medical University, Shanghai, China; ^4^ Department of Marine Biomedicine and Polar Medicine, Naval Medical Center of People’s Liberation Army (PLA), Naval Medical University, Shanghai, China

**Keywords:** heart failure, cardiotoxicity, autophagy, empagliflozin, sunitinib

## Abstract

**Background:** Sodium–glucose cotransporter-2 (SGLT2) inhibitors have been shown to decrease the adverse cardiac events and risks of cardiovascular mortality among patients with or without diabetes, which has made these drugs promising treatment options for patients with chronic heart failure. Cardiac dysfunction is a common and severe side effect induced by cancer chemotherapies, which seriously affects the prognosis and life quality of tumor patients. However, it is not clear whether SGLT2 inhibitors have cardiovascular benefits in patients with cancer chemotherapy–related cardiac dysfunction. We aimed to determine whether empagliflozin (EMPA), an SGLT2 inhibitor, has a protective role against sunitinib (SNT)-induced cardiac dysfunction in a mouse model.

**Methods:** Male C57BL/6J mice were randomized into control (control, *n* = 8), empagliflozin (EMPA, *n* = 8), sunitinib (SNT, *n* = 12), or sunitinib and empagliflozin coadministration (SNT + EMPA, *n* = 12) groups. EMPA, SNT, or SNT-combined EMPA was given *via* oral gavage for consecutive 28 days. Cardiovascular functions and pathological changes were examined, and the underlying mechanisms of EMPA’s effects were investigated in H9c2 cardiomyocytes.

**Results:** Mice in the SNT group exhibited dramatically elevated blood pressure (systolic blood pressure [SBP] 134.30 ± 6.455 mmHg vs. 114.85 ± 6.30 mmHg) and impaired left ventricular function (left ventricular ejection fraction [LVEF] 50.24 ± 3.06% vs. 84.92 ± 2.02%), as compared with those of the control group. However, EMPA could ameliorate SNT-induced cardiotoxicity, both in terms of SBP (117.51 ± 5.28 mmHg vs. 134.30 ± 6.455 mmHg) and LVEF (76.18 ± 5.16% vs. 50.24 ± 3.06 %). In H9c2 cardiomyocytes, SNT-induced cardiomyocyte death and cell viability loss as well as dysfunction of adenosine 5’-monophosphate–activated protein kinase–mammalian target of rapamycin (AMPK-mTOR) signaling–mediated autophagy were restored by EMPA. However, these favorable effects mediated by EMPA were blocked by the inhibition of AMPK or autophagy.

**Conclusion:** EMPA could ameliorate SNT-induced cardiac dysfunction *via* regulating cardiomyocyte autophagy, which was mediated by the AMPK-mTOR signaling pathway. These findings supported that SGLT2 inhibitor therapy could be a potential cardioprotective approach for cardiovascular complications among patients receiving SNT. However, these favorable effects still need to be validated in clinical trials.

## Background

Sodium–glucose cotransporter-2 (SGLT-2) inhibitors have been recently used in the treatment of type 2 diabetes mellitus (T2DM), and these agents could lower blood glucose concentration by enhancing urinary glucose excretion, as well as maintain long-term effect on the blood HbA1c level (Zinman et al., 2015; Neuen et al., 2018; Wiviott et al., 2019). Empagliflozin (EMPA), a potent and highly selective SGLT2 inhibitor, has been approved for the treatment of T2DM in 2015 (Fala, 2015). In fact, in addition to its favorable role in lowering serum glucose, recently published EMPA-REG OUTCOME study demonstrated that in patients with T2DM and high cardiovascular disease (CVD) risk, EMPA could decrease the adverse cardiac events by 14% and reduce cardiovascular mortality by 38% (Zinman et al., 2015). Similarly, it has been proposed that SGLT2 inhibitors have several direct or indirect protective effects on the progression of heart failure (HF) (Berliner and Bauersachs, 2017). Dapagliflozin and canagliflozin have also been reported to reduce the primary composite endpoint of cardiovascular death or hospitalization for heart failure (Neuen et al., 2018; Wiviott et al., 2019). These favorable cardioprotective effects of SGLT2 inhibitors independent of blood glucose controls have made them to be a potential treatment for chronic heart failure patients with or without T2DM. However, the mechanisms underlying behind these effects remain elusive.

Over the last decades, the survival rate of cancer patients has increased dramatically, which was largely driven by the development of novel cancer therapeutic strategies (Zamorano et al., 2016). Despite these advancements, cardiotoxicity, especially cardiac dysfunction or even heart failure, became the most common observed adverse effect of cancer treatments (Curigliano et al., 2016). Hence, the emerging interdisciplinary field of cardio-oncology is rapidly growing (Campia et al., 2019). With the development of cardio-oncology, cardiotoxicity was observed not limited to just anthracyclines; instead, novel targeted chemotherapeutic drugs, such as tyrosine kinase inhibitors (TKIs) and proteasome inhibitors (PIs), have been confirmed with a variety of cardiac complications, including heart failure, vascular disease, accelerated hypertension, and arrhythmias (Zamorano et al., 2016). Sunitinib (SNT) is a small molecule, multi-targeted tyrosine kinase inhibitor, and widely used as an effective anticancer drug for the treatment of advanced renal cell carcinomas (Motzer et al., 2007), gastrointestinal stromal tumors (Reichardt et al., 2015), and advanced pancreatic neuroendocrine tumors (Blumenthal et al., 2012). Unfortunately, current strategies based on SNT were shown to be associated with serial severe adverse effects, such as the development of hypertension and cardiac dysfunction, which would significantly contribute to poor long-term prognosis of such patients (Di Lorenzo et al., 2009; Narayan et al., 2017). However, there is still a lack of effective treatment for SNT-induced cardiovascular dysfunction. Therefore, it is extremely imperative to develop novel strategies to prevent the SNT-induced cardiotoxicity without compromising its anticancer efficacy.

For this reason, we sought to determine the potential protective effects of EMPA on SNT-induced cardiotoxicity and explore the underlying mechanisms. In this context, an *in vivo* study based on a mouse model of cardiac dysfunction induced by SNT and an *in vitro* study in SNT-treated H9c2 cardiomyocytes with or without EMPA were implemented, respectively. Data from our study showed that EMPA could ameliorate SNT-induced hypertension and left ventricular dysfunction in mice, and alleviate SNT-induced H9c2 cardiomyocyte viability loss *via* regulation of adenosine 5’-monophosphate–activated protein kinase–mammalian target of rapamycin (AMPK-mTOR) signaling–mediated autophagy. These findings indicated that EMPA could be a potential cardioprotective approach for prevention and treatment of SNT-induced cardiotoxicity in clinic. However, these favorable effects still need to be validated in clinical trials.

## Methods

### Animals Ethics Statement

All animal experiments were approved and performed in accordance with the guidelines of the Institutional Animal Care and Use Ethics Committee of Naval Medical University and the Guide for the Care and Use of Laboratory Animals of the National Institutes of Health (United States). All mice were bred and maintained in climate-controlled, specific, pathogen-free conditions with a 12-h light/dark cycle and free access to water and chow diet.

### Animal and Treatment

Wild-type C57BL/6J mice were purchased from the SLAC Laboratory (Shanghai SLAC Laboratory Animal Co., Ltd., China). Forty 8-week-old male mice were used in the study, and they were randomized into groups treated with vehicle (Control, *n* = 8), empagliflozin (EMPA, *n* = 8), sunitinib (SNT, *n* = 12), or sunitinib plus empagliflozin (SNT + EMPA, *n* = 12). SNT (HY-10255A, MedChemExpress, Shanghai, China) and EMPA (HY-15409, MedChemExpress, Shanghai, China) were both diluted in 5% DMSO in order to be administered to the mice. Dosage of SNT was 40 mg/kg/°day (Chintalgattu et al., 2013) and EMPA was 10 mg/kg/day (Andreadou et al., 2017; Yang et al., 2019), both of which were given to mice via oral gavage for 28 consecutive days. The overall study protocol shows more detail in the additional file (Supplementary Figure S1).

### Blood Pressure Measurement

Arterial blood pressure was measured noninvasively using a tail-cuff system (ALC-NIBP, Alcott, Shanghai, China) at baseline; days 7, 14, and 21; and at the endpoint. Blood pressure parameters including systolic blood pressure (SBP), mean blood pressure (MBP), diastolic blood pressure (DBP), and heart rate (HR) were recorded. An average value of measurements was taken from 10 consecutive measured cycles in each mouse (Zhao et al., 2011).

### Fasting Blood Glucose Measurement

Fasting blood glucose was determined at baseline; days 7, 14, and 21; and at the end of the experiment using a glucometer (Yuwell-590, Jiangsu, China).

### Echocardiographic Assessment

All mice underwent echocardiographic measurements at baseline and day 28 after treatment. Mice were anesthetized by spontaneous inhalation and maintained under general anesthesia with 1–2% isoflurane. Transthoracic echocardiography was performed by a high-resolution ultrasound imaging system (VINNO 6, Vinno Corporation, Suzhou, China) with a 23 MHz probe. M-mode recordings were obtained from the parasternal short axis views. The internal dimensions of left ventricular (LV) cavity, LV internal diameter in diastole (LVIDd), LV internal diameter in systole (LVIDs), LV ejection fraction (LVEF), fractional shortening (FS), interventricular septal thickness in diastole (IVSd), interventricular septal thickness in systole (IVSs), LV posterior wall thickness in diastole (LVPWd), and LV posterior wall thickness in systole (LVPWs) were measured to evaluate the LV systolic function. After an apical four-chamber view was obtained, the pulsed wave Doppler (PWD) cursor was placed between the tips of the open mitral leaflets, and PWD was used to study the flow in this area. LV diastolic functions were determined by using the mitral valve study mode to calculate the E wave, A wave, E/A ratio, deceleration time (DT), and isovolumetric relaxation time (IVRT).

### Coronary Flow Reserve Measurement

Coronary flow reserve (CFR) was used as an index of coronary microvascular function and measured as previously described (Hartley et al., 2008). Briefly, after the animal was anesthetized with 1% isoflurane, a pulsed Doppler ultrasound probe tip was placed at the left side of its chest and stabilized by a micromanipulator (model MM3-3, World Precision Instruments). The probe was fixed in a position when peak coronary velocities were identified. As a coronary vasodilator stimulus, basal (with 1% isoflurane) and a dilated (induced with 2.5% isoflurane) coronary flow were recorded and analyzed using a Doppler ultrasound system (Indus Doppler flow velocity system). Ratios of peak velocities at dilated and a basal state were reported as CFR.

### Enzyme-Linked Immunosorbent Assay

Measurements for plasma cardiac Troponin T (cTnT) and N-terminal pro–B-type natriuretic peptide (NT-proBNP) were performed with enzyme-linked immunosorbent assay (ELISA) kits (WESTANG BIO-TECH CO.LTD., Shanghai, China) and detected using an absorbance microplate reader (BioTek, United States) according to instructions.

### Histopathology and Immunohistochemistry

Mice hearts were fixed with 4% paraformaldehyde in phosphate-buffered saline at room temperature for 24 h and embedded in paraffin. Cardiac tissue sections (5 μm) were stained with hematoxylin and eosin (H&E), Masson’s trichrome (MT), and FITC-conjugated wheat germ agglutinin (WGA) according to the standard procedure. Microvessels were visualized through the expression of CD31 in cardiac tissue sections by immunohistochemistry. The sections were incubated with 10% goat blocking serum for 30 min at room temperature, then with a primary antibody against CD31 (GB11063-1, Servicebio, Wuhan, China) overnight at 4°C, and finally with a HRP-conjugated secondary antibody. Rinsed sections were counterstained with hematoxylin. Bright-field images were acquired by using light microscopy (Olympus, BX41, Japan). Cardiac fibrosis, cardiomyocyte size, and myocardial capillary density were quantified by Image-Pro Plus 6.0 software (Media Cybernetics, Sarasota, Florida, United States).

### Cell Culture

Embryonic rat cardiomyocyte–derived cell line H9c2 was obtained from American Type Culture Collection (ATCC, Manassas, VA, United States). Cells were cultured in DMEM supplemented with 10% fetal bovine serum (FBS) and 1% penicillin (100°U/ml)/streptomycin (100°U/°ml) at 37°C in a 5% CO_2_ atmosphere. When H9c2 cells reached about 80–90% confluence, the cells were seeded in a 96-well plate or in a 6-well plate at 4 × 10^3^/well or 6 × 10^4^/well and left in culture for 24 h to perform cell viability assay, Hoechst staining, and autophagic flux analysis.

### Cell Viability Assay

Cell viability assay of H9c2 cardiomyocytes was conducted using Cell Counting Kit-8 (CCK-8, Dojindo, Kumamoto, Japan) according to the standard procedure. For assessment of SNT-induced cell viability changes, H9c2 cells were planted onto 96-well and treated with serial concentrations of SNT (0, 1, 2, 5, 10, and 20 μM) for 48 h. IC50 of SNT was calculated using GraphPad Prism 8.0 (GraphPad, San Diego, CA, United States). For assessment of the protective effect of EMPA on SNT-induced cell viability loss, H9c2 cells were co-treated with SNT (5 μM) and serial concentrations of EMPA (50, 100, 500, and 1, 000 nM) for 48 h. To confirm the role of AMPK-mTOR signaling pathway–mediated autophagy in the protection of EMPA against SNT-induced cell viability loss, H9c2 cells were pretreated with AMPK inhibitor compound C (10 μM) or autophagy inhibitor bafilomycin A1 (50 nM) prior to SNT or SNT plus EMPA treatment for 12 h. Optical density values at 450 nm were measured, and relative cell viability was calculated as viability vs. control. Each CCK-8 experiment was repeated 6 times.

### TUNEL Staining

Mice heart sample cryosections or H9c2 cells were stained using One Step TUNEL Apoptosis Assay Kit (C1086, Beyotime Institute of Technology, Shanghai, China) and counterstained with DAPI to visualize nuclei. Images were photographed using confocal laser scanning microscopy (Zeiss, Oberkochen, Germany) and quantified using ImageJ software (National Institutes of Health, United States).

### Hoechst Staining

Cell apoptosis was quantified by Hoechst 33, 258 staining (Beyotime Biotechnology, Inc., Shanghai, China) according to the instructions. The morphologic changes in the apoptotic cell nuclei were detected using a fluorescence microscope (Olympus, BX53; Melville, NY, United States).

### Autophagic Flux Measurements in H9c2 Cardiomyocytes

In order to monitor the autophagic flux in H9c2 cells, mCherry-green fluorescent protein (GFP)-tagged LC3 adenovirus (Ad-mCherry-GFP-LC3) was used as previously reported (Yang et al., 2019). GFP is sensitive to the acidic environment and will be quenched in the acidic lysosomal, while mCherry is more stable. As a consequence, autophagosomes (yellow) can be distinguished from autolysosomes (red), and flux can be tested directly (Mizushima et al., 2010). H9c2 cells were planted in a 6-well plate and transiently transfected with Ad-mCherry-GFP-LC3 (MOI = 40) for 24 h. Then, the medium was replaced, and H9c2 cells were treated by SNT with or without EMPA. After 48 h incubation, the cells were imaged under a fluorescent microscope. At least 6 cells from each group were analyzed for the number of autophagic puncta.

### Western Blot Analysis

At the end of the experimental period, differently treated mice hearts or protein samples of H9c2 cells were completely lysed in ice-cold RIPA and sent to centrifuge, and protein concentration was measured using the BCA Protein Assay Kit (Beyotime Institute of Technology, Shanghai, China). A total of 20–40 μg protein per lane were separated by sodium dodecyl sulfate–polyacrylamide gel electrophoresis on a 10% or 15% gel and transferred onto a polyvinylidene difluoride (PVDF) membrane (EMD Millipore, Billerica, MA, United States). Subsequently, blocking was performed by 5% bovine serum albumin (BSA) in Tris-buffered saline Tween 20 (TBST) for 2 h at room temperature and then washed three times with TBST. The membranes were incubated with primary antibodies against LC3A/B (#12741, Cell Signaling Technology, United States, diluted to 1:1000), p62 (#5114, Cell Signaling Technology, United States, diluted to 1:1000), mTOR (380,411, ZenBio, Chengdu, China, diluted to 1:1000), *p*-mTOR (381,557, ZenBio, Chengdu, China, diluted to 1:1000), AMPKα (#2532, Cell Signaling Technology, United States, diluted to 1:1,000), *p*-AMPKα (#2531, Cell Signaling Technology, United States, diluted to 1:1,000), Akt (#9272, Cell Signaling Technology, United States, diluted to 1:1,000), *p*-Akt (# 4060S, Cell Signaling Technology, United States, diluted to 1:1,000), cleaved caspase-3 (#9661, Cell Signaling Technology, United States, diluted to 1:1,000), and Bax (200958, ZenBio, Chengdu, China, diluted to 1:1000) at 4°C temperature for overnight. The membranes were then incubated with the indicated secondary antibodies (goat against rabbit or mouse, diluted to 1:5,000) for 2 h at room temperature. The protein bands were visualized using a Tanon Imaging System (version 5200, Tanon Science & Technology Co., Ltd., Hi-tech Park, Shanghai, China) and analyzed quantitatively using ImageJ software (National Institutes of Health, Bethesda, MD).

### Statistical Analysis

All values were analyzed with GraphPad Prism software 8.0 (San Diego, CA, United States) and shown as mean ± SEM. SBP, DBP, MBP, and HR acquired by tail-cuff in conscious mice among different groups were analyzed by repeated measurement analysis of variance (ANOVA) followed by Tukey’s post hoc test. One-way ANOVA followed by Bonferroni’s post hoc test was used for the other multiple comparisons in this study. *p*-value less than 0.05 was considered statistically significant.

## Results

### Empagliflozin Attenuated SNT-Induced Hypertension and Cardiac Dysfunction

**FIGURE 1:**
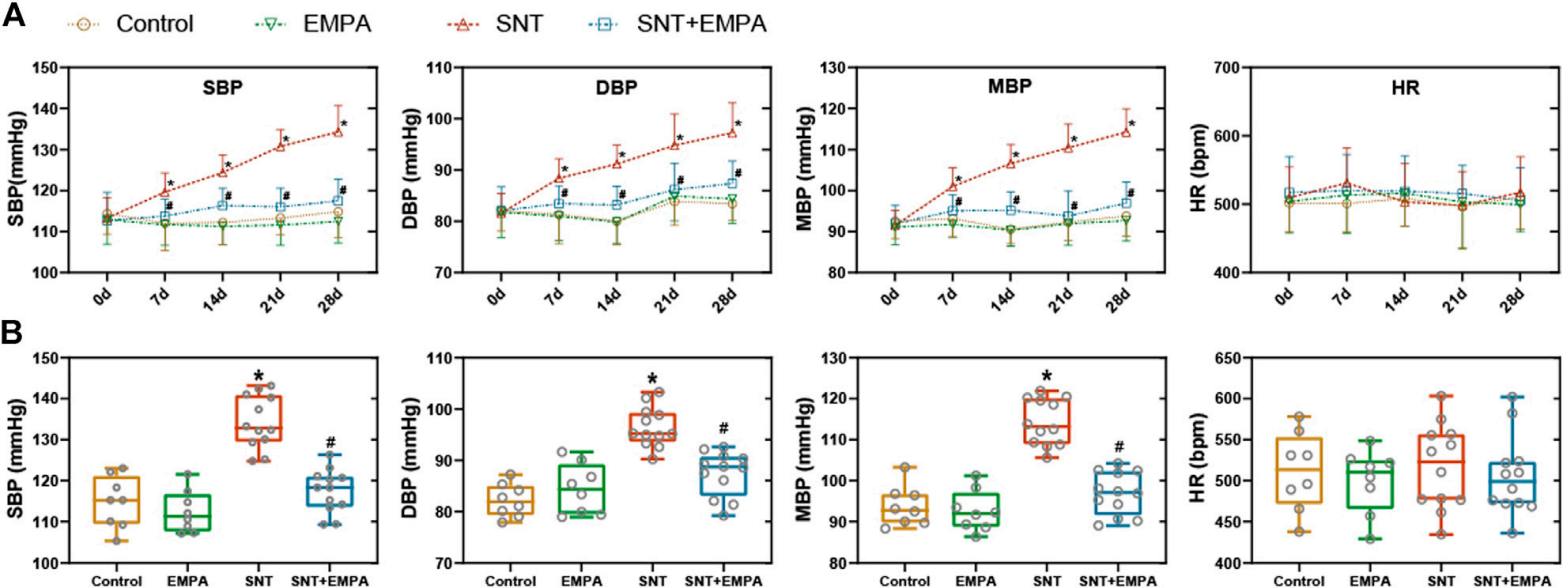
Effects of EMPA on blood pressure in SNT-treated mice. **(A)** Changes of blood pressure (SBP, DBP, and MBP) and HR during the modeling process among different groups, *n* = 8 or 12 per group. **(B)** Blood pressure (SBP, DBP, and MBP) and HR at the study endpoint among different groups, *n* = 8 or 12 per group. *p < 0.05 vs. control, #*p* < 0.05 vs. SNT.

**FIGURE 2:**
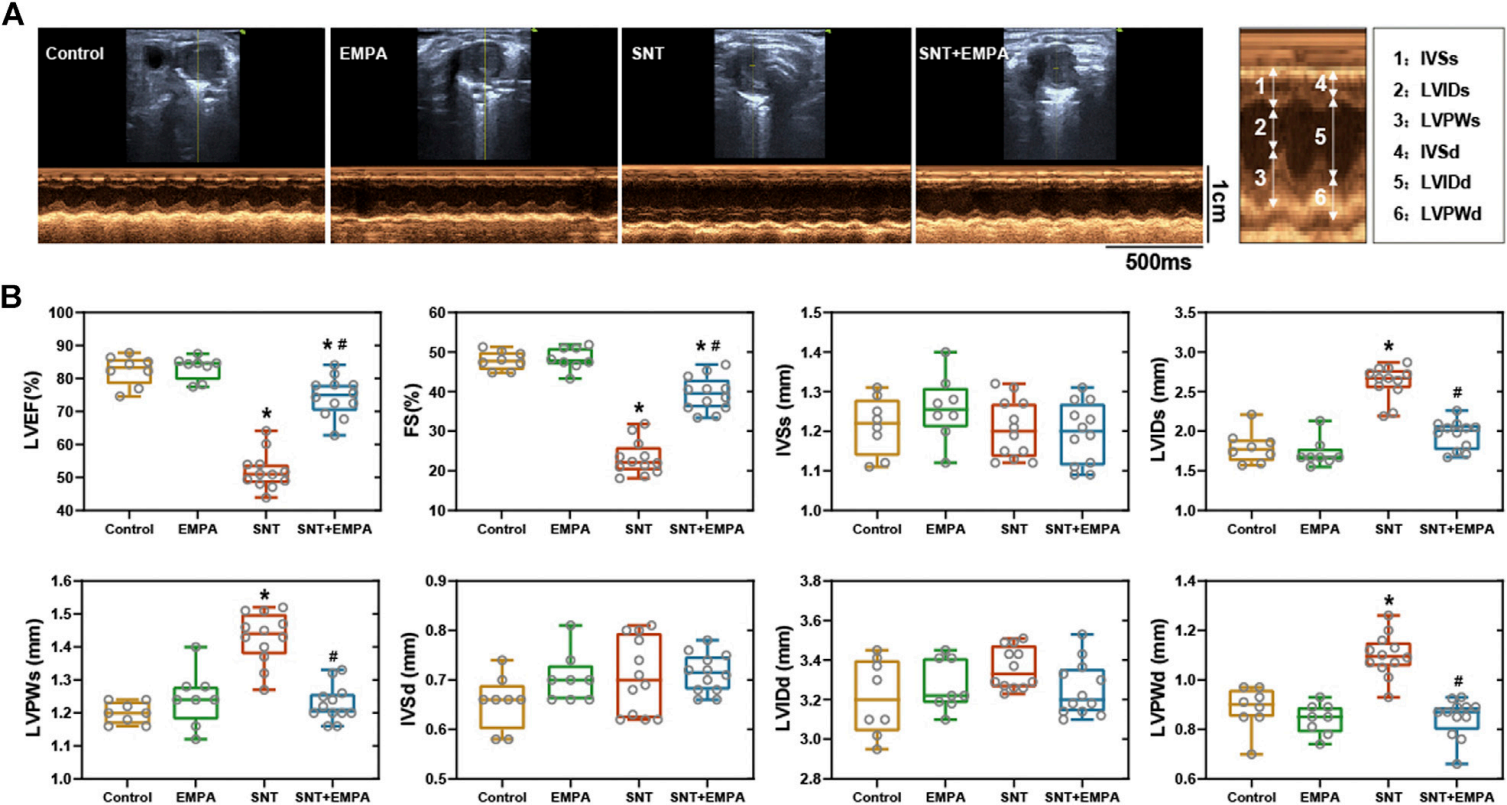
Effects of EMPA on left ventricular systolic function in SNT-treated mice. **(A)** Representative recordings of M-mode echocardiography in a parasternal short axis view at the study endpoint among different groups, *n* = 8 or 12 per group. **(B)** Left ventricular systolic function parameters evaluated from M-mode images at the study endpoint among different groups, *n* = 8 or 12 per group. *p < 0.05 vs. control, #*p* < 0.05 vs. SNT.

**FIGURE 3:**
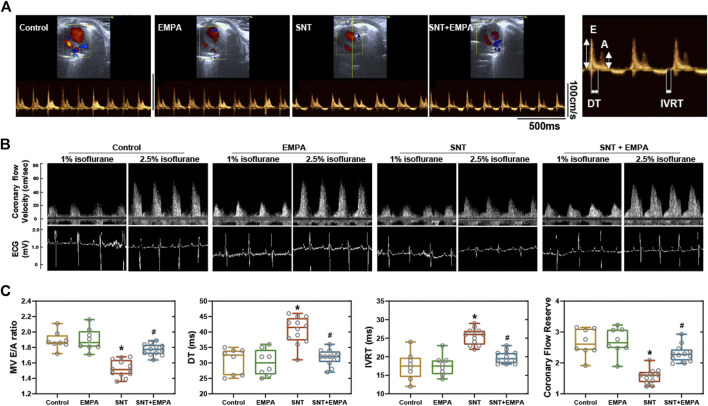
Effects of EMPA on left ventricular diastolic function and coronary flow reserve in SNT-treated mice. **(A)** Representative recordings of mitral valve inflow by pulsed wave Doppler in an apical four-chamber view at the study endpoint among different groups, *n* = 8 or 12 per group. **(B)** Left ventricular diastolic function parameters evaluated from mitral valve flow spectrum among different groups, *n* = 8 or 12 per group. **(C)** Representative ultrasound tracings of dilated (induced with 2.5% isoflurane) and basal (with 1% isoflurane) coronary flow at the study endpoint. **(D)** Quantification of coronary flow reserve (dilated/basal flow) among different groups, *n* = 8 or 12 per group. *p < 0.05 vs. Control, #*p* < 0.05 vs. SNT.

All mice had a structurally and functionally normal heart before experiments, and no difference of body weight, blood glucose, and blood pressure was shown among these groups at the baseline (Supplementary Table S1). To investigate the effects of EMPA on SNT-induced cardiotoxicity, we evaluated the cardiovascular parameters of differently treated mice at the study endpoint. As shown in Figure 1, SNT-treated mice exhibited dramatically elevated SBP, DBP, and MBP, without affecting HR, as compared with those from the control group, suggesting that SNT would induce hypertension in mice. However, such pressure-increasing effects of SNT could be dramatically alleviated by EMPA. Furthermore, echocardiographic data showed impaired LVEF% and FS%, and increased LVIDs, LVPWs, and LVPWd (Figures 2A,B), as well as decreased MV E/A ratio and prolonged DT and IVRT (Figures 3A,C) in SNT-treated mice compared with those from the control group, indicating that SNT would induce left ventricular systolic and diastolic dysfunction, while coadministration of EMPA could significantly reverse such adverse events. In order to determine the effects of SNT on coronary microvascular function, coronary flow reserve (CFR), an index of coronary microvascular function, was measured by a pulsed Doppler system. Decreased CFR was observed in SNT-treated mice, which suggested that SNT treatment would induce coronary microvascular dysfunction (Figures 3B,C). Nevertheless, EMPA could effectively reverse the SNT-induced CFR decrease (Figure 3C). And SNT could significantly increase plasma cTnT (1468.87 ± 335.18 vs. 276.28 ± 106.15 pg/ml) and pro-BNP levels (17.81 ± 5.52 vs. 11.38 ± 1.12 pg/ml) compared with the control group, which could be alleviated by EMPA (all *p* < 0.05; Table 1). However, no obvious statistical differences were observed in terms of body weight, heart weight, lung weight, and blood glucose between these groups (Table 1; Supplementary Figure S2). And no significant difference concerning cardiac fibrosis, cardiomyocyte size, and microvascular density was found among different groups (Supplementary Figure S3). Taken together, EMPA treatment could alleviate SNT-induced hypertension and cardiac dysfunction.

**TABLE 1:** Weight, glucose, and blood parameters at the study endpoint.

	Control (*n* = 8)	EMPA (*n* = 8)	SNT (*n* = 12)	SNT + EMPA (*n* = 12)
Body weight (g)	24.33 ± 1.14	23.59 ± 1.17	23.22 ± 1.18	23.68 ± 1.17
Heart weight (mg)	116.25 ± 10.61	112.50 ± 10.35	115.83 ± 13.11	112.5 ± 9.65
Lung weight (mg)	136.25 ± 10.61	132.50 ± 8.86	135.83 ± 9.96	131.67 ± 10.30
Heart weight/Body weight (mg/g)	4.77 ± 0.27	4.76 ± 0.32	4.98 ± 0.44	4.75 ± 0.30
Lung weight/Body weight (mg/g)	5.60 ± 0.24	5.62 ± 0.25	5.86 ± 0.45	5.56 ± 0.21
Heart weight/tibial length (mg/mm)	6.22 ± 0.57	6.12 ± 0.52	6.37 ± 0.63	6.22 ± 0.44
Blood glucose (mmol/L, fasting)	5.64 ± 0.63	5.46 ± 0.66	5.52 ± 0.64	5.35 ± 0.66
Plasma cTnT (pg/ml)	276.28 ± 106.15	234.88 ± 164.93	1,468.87 ± 335.18^*^	724.55 ± 209.28^#^
Plasma NT-proBNP (pg/ml)	11.38 ± 1.12	11.58 ± 1.74	17.81 ± 5.52^*^	11.06 ± 3.93^#^

Data are expressed as the mean ± SD. cTnT: cardiac troponin T, NT-proBNP: N-terminal pro–B-type natriuretic peptide. *p* <0.05 vs. control, ^#^ *p* < 0.05 vs. SNT.

### Empagliflozin Ameliorated SNT-Triggered Cardiomyocyte Death and Cell Viability Loss

**FIGURE 4:**
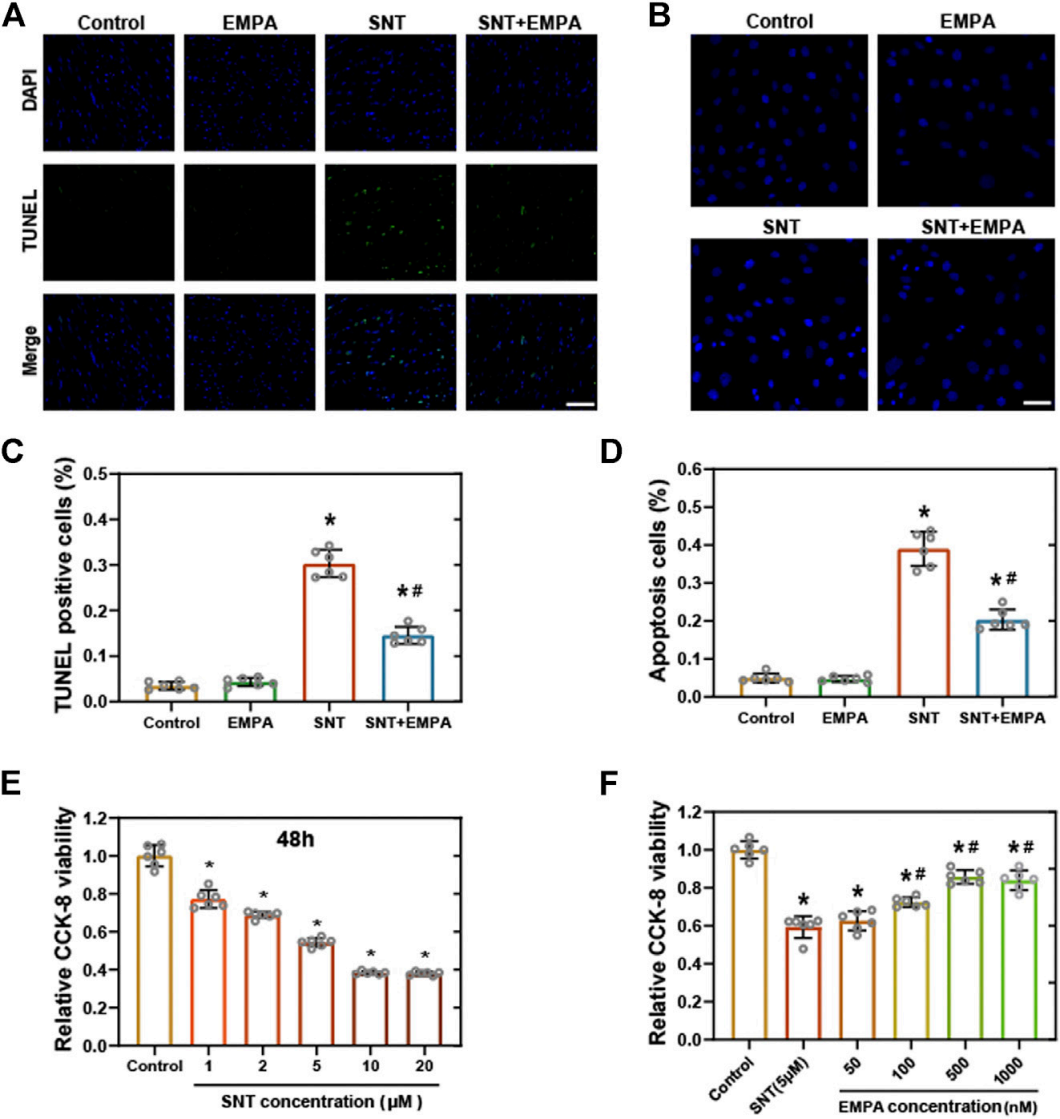
EMPA ameliorated SNT-triggered cardiomyocyte death and cell viability loss. **(A)** Representative images of TUNEL positive cells in mice hearts at the study endpoint among different groups and **(C)** statistical analysis, *n* = 6 per group, bar = 50 μm. **(B)** Representative images of Hoechst 33,258 staining in H9c2 cells with different treatments and **(D)** statistical analysis, *n* = 6 per group, bar = 50 μm. (E) CCK-8 viability assay of H9c2 cardiomyocytes treated with vehicle or different concentrations of SNT (ranging from 1 to 20 μM), *n* = 6 per group. **(F)** CCK-8 viability assay of H9c2 cardiomyocytes treated by vehicle, SNT (5 μM), or SNT plus EMPA (ranging from 50 to 1,000 nM), *n* = 6 per group. *p < 0.05 vs. control, #*p* < 0.05 vs. SNT.

We then examined cardiomyocyte death in mice and cell viability in H9c2 cells among different groups. As shown by TUNEL assay, mice treated by SNT showed more positive cells ratio than those of the control group, indicating SNT would increase cardiomyocyte death (*p* < 0.001), whereas such effects could be reversed by EMPA (Figures 4A,C). Hoechst 33, 258 staining in H9c2 cells also suggested more cell death in the SNT group and improved cell death rate in the SNT + EMPA group (Figures 4B,D). In H9c2 cardiomyocytes, SNT (ranging from 1 to 20 μM) was shown to induce decreased cell viability in a dose-dependent manner, with IC_50_ of 6.71 μM (Figure 4E). However, SNT (5 μM) co-treatment with EMPA (ranging from 100 to 1,000 nM) significantly alleviated such cell viability decline (Figure 4F). Due to increased TUNEL positive cells and decreased cardiomyocyte viability induced by SNT, expression changes of apoptotic markers were investigated, such as cleaved caspase-3 and Bax both in mice and H9c2 cells. However, no significant alterations were found in these apoptotic markers among different groups (Supplementary Figure S4), implying that the classical apoptosis pathways might not be involved with SNT-induced cardiomyocyte death.

### Empagliflozin Reversed SNT-Induced Cardiomyocyte Autophagic Inhibition

**FIGURE 5:**
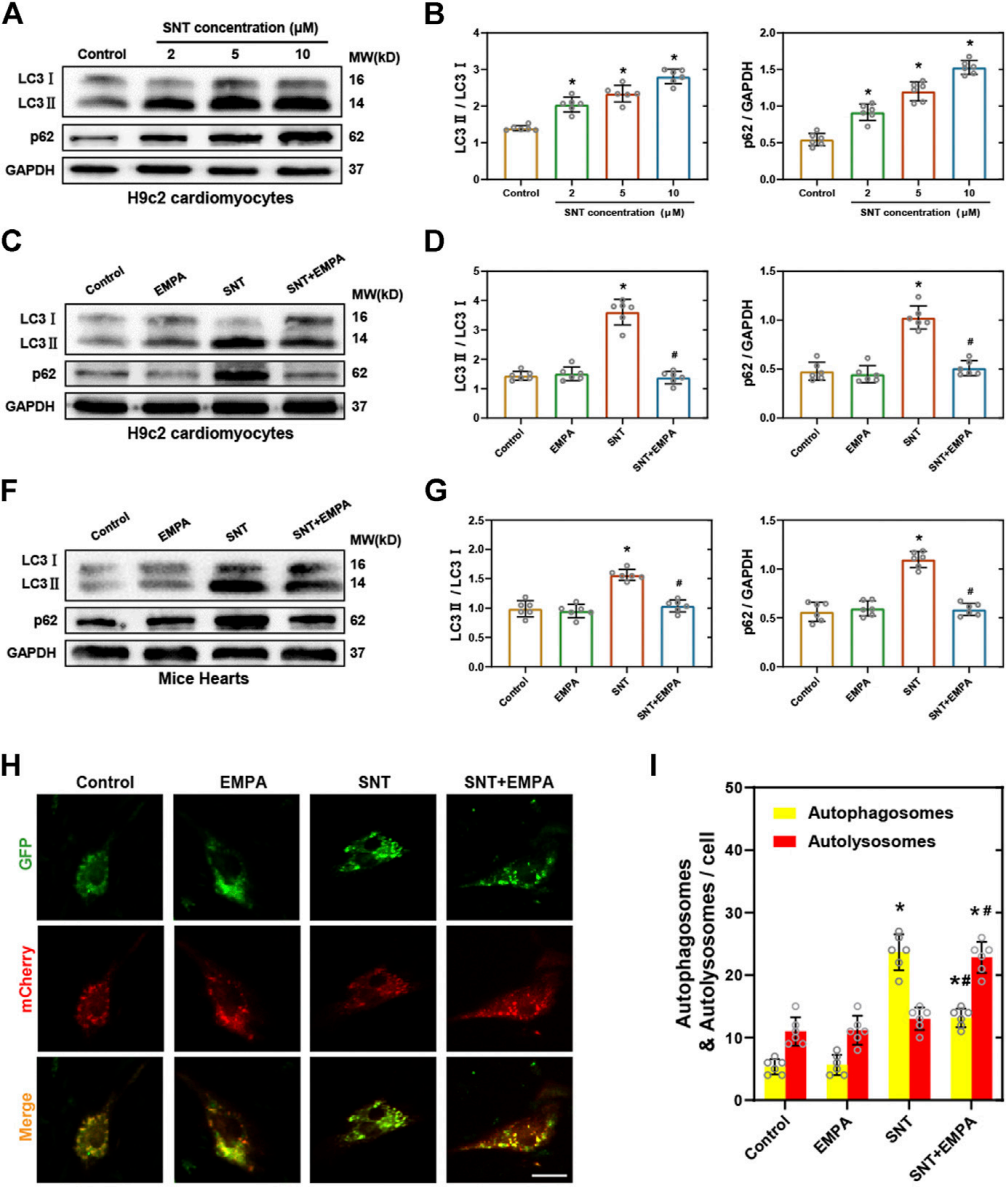
EMPA reversed SNT-induced autophagic inhibition. **(A)** Representative Western blots of LC3-II to LC3-I ratio and p62 in H9c2 cardiomyocytes treated with different concentrations of SNT. **(B)** Relative protein expression quantification of LC3-II to LC3-I ratio and p62 as illustrated in **(A)**, *n* = 6 per group. **(C)** Representative Western blots of LC3-II to LC3-I ratio and p62 in H9c2 cardiomyocytes treated by vehicle, EMPA, SNT, or SNT plus EMPA. **(D)** Relative protein expression quantification of LC3-II to LC3-I ratio and p62 as illustrated in **(C)**, *n* = 6 per group. **(E)** Representative Western blots of LC3-II to LC3-I ratio and p62 in mice hearts treated by vehicle, EMPA, SNT, or SNT plus EMPA. **(F)** Relative protein expression quantification of LC3-II to LC3-I ratio and p62 as illustrated in **(E)**, *n* = 6 per group. **(G)** Representative fluorescence images of mCherry-GFP-LC3 adenovirus-transfected H9c2 cells treated by vehicle, EMPA, SNT, or SNT plus EMPA. **(H)** Statistical analysis of yellow (autophagosome) and red (autolysosome) puncta per cell, *n* = 6 cells per group, bar = 20 μm *p < 0.05 vs. control, #*p* < 0.05 vs. SNT.

Autophagy, an evolutionarily conserved cellular degradation process, has gained increasing recognition in recent years for its vital role in both cell survival and cell death (Mizushima and Levine, 2020). Previously, autophagy has been shown to exert a critical role in the anticancer efficacy of SNT (DeVorkin et al., 2017; Dyczynski et al., 2018; Thakur et al., 2018). However, the exact mechanism of autophagy in SNT-induced cardiomyocyte death remains to be elucidated. The autophagic flux upon SNT challenge in both H9c2 cells and mice hearts was explored. Conversion of LC3-I into LC3-II is considered as a marker of autophagic vesicles, while p62 is a selective autophagy cargo receptor, involving with the degradation of autophagy substrates. As shown in Figure 5, both the ratio of LC3-II to LC3-I and the expression of p62 were significantly upregulated by SNT in H9c2 cardiomyocytes in a concentration-dependent manner (Figures 5A,B), and this change was restored by EMPA (Figures 5C,D). *In vivo* experiments also found that SNT increased the ratio of LC3-II to LC3-I and the expression of p62 in mouse myocardium, whereas EMPA could restore such changes (Figures 5E,F). To further confirm EMPA’s effects on SNT-induced autophagy, H9c2 cardiomyocytes were transfected with mCherry-GFP-LC3 adenovirus to observe autophagic vesicles. As a result, SNT could trigger a dramatic accumulation of autophagosomes (yellow puncta), with no alteration of autolysosomes (red puncta) in H9c2 cardiomyocytes, and EMPA reversed SNT’s effects with much more autolysosomes and less autophagosomes (Figures 5G,H). Taken together, we deduced that autophagic flux within cardiomyocytes may be inhibited at the late stage in SNT-induced cardiotoxicity, while EMPA might improve the autophagic flux and make it fluent again to exert an anti-cardiotoxic effect against SNT.

### Empagliflozin Restored SNT-Induced Dysfunction of the AMPK-mTOR Signaling Pathway Both *In Vitro* and *In Vivo*.

**FIGURE 6:**
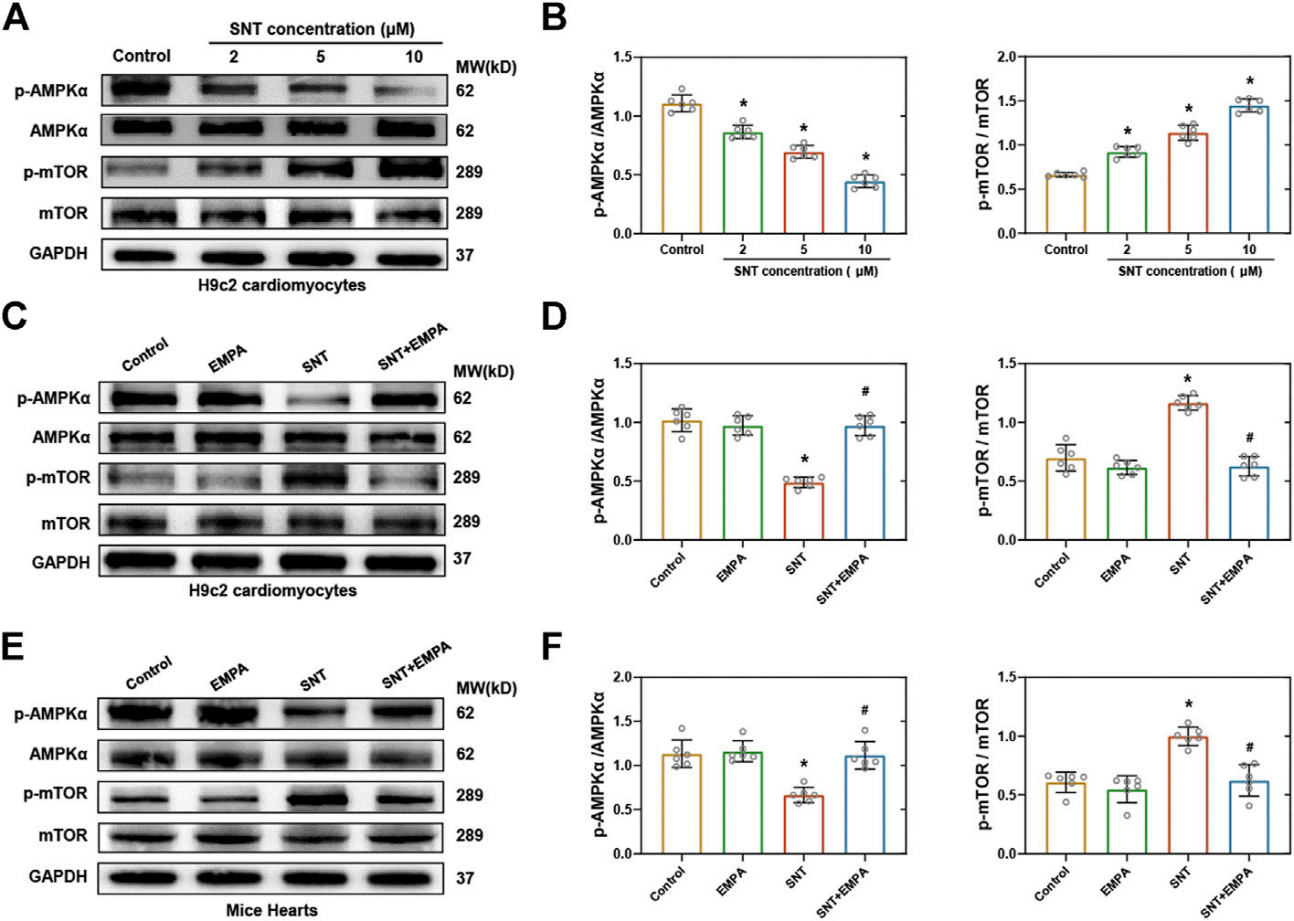
EMPA restored SNT-induced dysfunction of the AMPK–mTOR signaling pathway both *in vitro* and *in vivo*. **(A)** Western blots of the AMPK–mTOR pathway in H9c2 cardiomyocytes treated with different concentrations of SNT. **(B)** Protein-level quantifications of phosphorylated AMPK to total AMPK and phosphorylated mTOR to total mTOR ratios, *n* = 6 per group. **(C)** Western blots of the AMPK–mTOR pathway in H9c2 cardiomyocytes treated by vehicle, EMPA, SNT, or SNT plus EMPA. **(D)** Protein-level quantifications of phosphorylated AMPK to total AMPK and phosphorylated mTOR to total mTOR ratios, *n* = 6 per group. **(E)** Western blots of the AMPK–mTOR pathway in mice hearts treated by vehicle, EMPA, SNT, or SNT plus EMPA. **(F)** Protein-level quantifications of phosphorylated AMPK to total AMPK and phosphorylated mTOR to total mTOR ratios, *n* = 6 per group. *p < 0.05 vs. control, #*p* < 0.05 vs. SNT.

Next, we explored the underlying molecular pathways that mediated EMPA’s regulation of SNT-induced autophagic dysfunction. The AMPK-mTOR axis has emerged as one of the major pathways regulating autophagy, and it is well known that promoting phosphorylation of AMPK will inhibit mTOR and activate autophagy (Kim et al., 2011). Former studies have revealed that SNT inhibits the phosphorylation of AMPK and causes cardiomyocyte cytotoxicity (Kerkela et al., 2009). Therefore, we examined the expression of AMPK-mTOR pathway changes in H9c2 cardiomyocytes and mice hearts when challenged by SNT. Our data showed that SNT significantly suppressed phosphorylation of AMPK, while it significantly increased phosphorylation of mTOR both *in vitro* and *in vivo* (Figures 6A–D). Nevertheless, these effects were ablated by EMPA (Figures 6C–F). In addition, we also investigated the alteration of the Akt-dependent autophagy pathway (another important pathway-regulating autophagy) in SNT-treated cardiomyocytes. However, no significant alterations were observed in Akt or *p*-Akt among different groups (Supplementary Figure S5), implying that the Akt-dependent autophagy pathway might not be involved in SNT-induced autophagic inhibition. Therefore, these findings demonstrated that dysfunction of the AMPK-mTOR signaling pathway may play an essential role in SNT-induced cardiomyocyte autophagic inhibition, whereas EMPA could regulate this pathway to improve cardiomyocyte autophagy.

### Inhibition of AMPK or Autophagy Blocked EMPA-Conferred Protection Against SNT-Induced Cardiotoxicity in Cardiomyocytes.

**FIGURE 7:**
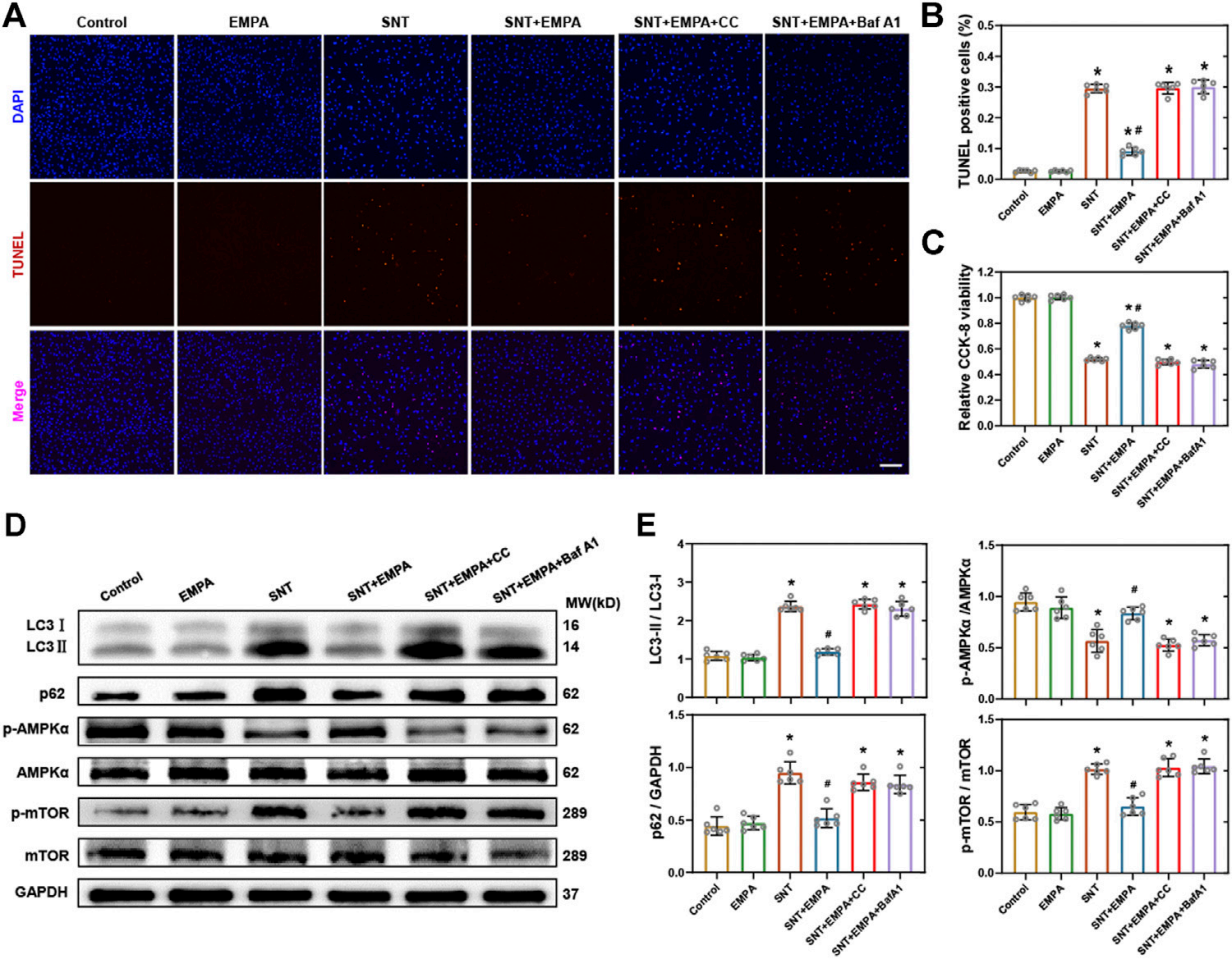
Inhibition of AMPK or autophagy-blocked EMPA-conferred protection against SNT-induced cardiotoxicity in cardiomyocytes. **(A)** Representative images of TUNEL positive cells in H9c2 cardiomyocytes treated by vehicle, EMPA, SNT, or SNT plus EMPA in combination with or without AMPK inhibitor compound C (CC) or autophagy inhibitor (Baf A1). **(B)** Statistical analysis among different groups, *n* = 6 per group, bar = 100 μm. (C) CCK-8 viability assay of H9c2 cardiomyocytes among different groups mentioned above, *n* = 6 per group. **(D)** Representative Western blots of the AMPK–mTOR–mediated autophagy pathway. **(E)** Protein-level quantifications of LC3-II to LC3-I, p62 to GAPDH, phosphorylated AMPK to total AMPK, and phosphorylated mTOR to total mTOR ratios, *n* = 6 per group. *p < 0.05 vs. control, #*p* < 0.05 vs. SNT.

To further confirm the role of the AMPK–mTOR signaling pathway–mediated autophagy in EMPA’s protecting against SNT-induced cardiotoxicity, we employed AMPK inhibitor compound C (CC) and autophagy inhibitor bafilomycin A1 (Baf A1) to study their relationships. Cardiomyocyte death, cell viability, AMPK-mTOR signaling cascade, and autophagy markers were assessed. H9c2 cardiomyocytes were pretreated with CC (10 μM) or Baf A1 (50 nM) prior to SNT or SNT plus EMPA treatment for 12 h, respectively. The TUNEL staining and cell viability assay quantification data revealed that SNT markedly increased the cardiomyocyte death rate and decreased cell viability, while EMPA attenuated SNT-induced cardiotoxicity (Figures 7A–C). Nevertheless, the EMPA’s protective effect on cardiomyocytes against SNT was blocked by CC or Baf A1 (Figures 7A–C). Furthermore, EMPA could restore SNT-induced autophagic inhibition as indicated by the decreased LC3II to LC3I ratio and expression of p62. But this restoration was offset by CC or Baf A1 (Figures 7D,E). Regarding AMPK-mTOR autophagy signaling cascade, our data showed that EMPA could ablate SNT-induced inhibition of AMPK and activation of mTOR, whereas this ablation was blocked by CC or Baf A1 (Figures 7D,E). Taken together, these results showed that EMPA’s anti-cardiotoxic effect against SNT was mainly depended on its regulation of AMPK–mTOR signaling pathway–mediated cardiomyocyte autophagy.

## Discussion

The clinical application of ample anticancer drugs has been hindered by their potential cardiotoxicity which dramatically compromises life qualities of cancer survivors. Hence, developing novel prevention and treatment strategies for chemotherapy-induced cardiotoxicity is imperatively urgent to improve overall prognosis and survival for cancer patients. EMPA, a selective inhibitor of SGLT2, has been reported to exert cardioprotective effects in several cardiovascular diseases, including diabetic cardiomyopathy (DCM) (Li et al., 2019), myocardial infarction (Andreadou et al., 2017), cardiorenal syndrome (Yang et al., 2019), and doxorubicin-induced heart failure (Oh et al., 2019). However, the effect of EMPA on SNT-induced cardiotoxicity remains obscure. This present study revealed that EMPA could ameliorate SNT-induced hypertension and cardiac dysfunction in a model of nondiabetic mice. Further histological examination showed a lower degree of cardiomyocyte death rate in mice treated with EMPA than in those treated by SNT. Molecular biological analysis demonstrated that SNT-induced cardiotoxicity was mediated *via* inhibition of late-stage autophagic flux, whereas this could be significantly improved by EMPA via the AMPK–mTOR pathway. Above all, these favorable effects of EMPA would provide a new insight for developing molecular treatment strategies for SNT-induced cardiotoxicity.

Extensive evidence has demonstrated that SNT is clinically effective in treating various types of solid tumors, such as renal cell carcinomas (Motzer et al., 2007) and gastrointestinal stromal tumors (Reichardt et al., 2015). However, SNT is associated with severe cardiovascular adverse effects, including hypertension, left ventricular dysfunction, and heart failure, which dramatically limits its clinical application (Di Lorenzo et al., 2009; Narayan et al., 2017). In fact, the most difficult challenge remains the lack of effective cardioprotective drugs. Hypertension is the most frequently encountered event of SNT-induced cardiovascular toxicity (Baek Moller et al., 2019). Ewer et al. performed a retrospective adjudication of comprehensive cardiovascular adverse events from two phase 3 trials enrolling 1090 patients and found that the incidence rate of hypertension was significantly higher with SNT than with placebo (Ewer et al., 2014). Therefore, most studies have been performed to identify potential cardioprotective drugs against SNT-induced cardiotoxicity aiming to keep blood pressure under control, such as macitentan and amlodipine (Lankhorst et al., 2014), and our study observed the same effects that SNT could significantly elevate blood pressure of mice, including SBP and DBP. However, such pressure-increasing effects of SNT could be dramatically alleviated by EMPA, which could be supported by data from previous studies that EMPA could significantly reduce the blood pressure in hypertension patients with or without diabetes (Heerspink et al., 2016; Kario et al., 2018). It was deduced that the pressure-decreasing effect of EMPA may be associated with its osmotic diuretic and natriuretic effects to reduce plasma volume. Although the urine volume and urinary sodium of the experimental animals were not recorded in this study, we observed that the sawdust litter in animal cages was changed more frequently in the EMPA group than in control or in the SNT only group during the modeling process. To some extent, this indicated that the antihypertensive effect of EMPA against SNT may be related to its diuretic effect, which may be probably independent of its antiapoptotic effects. However, the exact mechanism of the BP-lowering effects of EMPA on mice treated by SNT still needs further investigations. Taken together, the above results suggested that EMPA might be a potential new agent for patients with SNT-induced hypertension.

In addition to favorable effects on blood pressure, EMPA also showed protective impact on LVF in our study, which was supported by a previous randomized controlled trial study in terms of death from cardiovascular causes and hospitalization for heart failure (Zinman et al., 2015). An *in vivo* study from Yang et al. (2019) also revealed that compared with rats with cardiorenal syndrome, LVEF was remarkably preserved in animals treated with EMPA. Previously, a clinical study suggested that SNT could induce ECG changes including conduction disturbances, change in QRS amplitude, ST segment depression or elevation, T wave changes, and QT prolongation (Schmidinger et al., 2008). To be honest, the ECG changes were not the preset indicators of observation in our study initially, although we observed that SNT could induce ECG modifications, and these changes seems to be prevented by EMPA (Figure 3B), when we performed the experiment of noninvasive coronary flow measurement using a pulsed Doppler system. However, the relationship between the altered ECG and cardiac dysfunction in this situation is still elusive. In addition, the ECG waveform displayed in this system was only used to confirm the diastolic and systolic stage of heart, in order to identify the Doppler signals of coronary flow. In other words, it is not a professional ECG monitoring equipment. On the other hand, although lacking direct clinical evidence supporting the antiarrhythmic effect of EMPA, a recent study suggested that EMPA could significantly ameliorate the QT prolongation induced by sotalol (a class III antiarrhythmic drug) in rats (Ozgur Baris et al., 2021). Consequently, further investigations about the ECG modifications induced by SNT and its contribution to LV dysfunction as well as EMPA’s antiarrhythmic effect should be using a professional ECG monitoring and analyzing equipment in a future study. The improvement of LVF resulting from EMPA would provide a preliminary basic for the potential application of EMPA to SNT-induced cardiac dysfunction for favorable cardiovascular outcomes.

To further investigate the potential mechanism for the protective effect of EMPA on SNT-induced cardiac dysfunction, we explored whether cardiomyocyte apoptosis was involved with the abnormal LVF. Our results showed that SNT could dramatically increase the death rate of cardiomyocytes both *in vivo* and *in vitro*, which is consistent with a previous study (Kerkela et al., 2009). However, we did not find significant alterations in terms of the expression of apoptosis-related proteins, neither in H9c2 cardiomyocytes nor in mice hearts. Yang et al. (2019) reported similar results that SNT showed no impact on the alterations of apoptotic markers in cardiac pericytes. These results made us speculate that the cardiomyocyte death induced by SNT might not be mediated *via* the classical apoptosis pathway but by other mechanisms.

Autophagy, an intracellular bulk degradation system that is highly conserved in eukaryotes, in which impaired cytoplasmic organelles, proteins, and macromolecules, will be wrapped by double-membraned autophagosomes and transported to lysosomes to complete degradation for the recycling of the breakdown products (Shibutani et al., 2015). In addition, autophagy has been generally considered as a survival mechanism, owing to the close correlation between its dysregulation and non-apoptotic cell death (Sciarretta et al., 2018). There exist basal levels of autophagy within cells in order to protect cells against various extracellular stresses, such as starvation or hypoxia (Glick et al., 2010). In general, conversion of the free cytoplasmic form of LC3-I into LC3-II is considered as an key indicator for degradation in the autophagic vesicles. Previous studies have reported that autophagy was associated with cellular sensitivity to SNT in different types of cancer cell (DeVorkin et al., 2017; Dyczynski et al., 2018; Thakur et al., 2018). Yang et al. also found the inhibition of autophagy plays a critical role in SNT-induced cardiac pericyte death (Yang et al., 2019b). However, recent studies about SNT-induced cardiomyocyte death have revealed contradictory results about the involvement of SNT in autophagy. Therefore, to determine the effect of SNT on autophagy in cardiomyocytes, we first examined the expression of LC3 following SNT treatment. As shown in results, SNT increased the ratio of LC3-II to LC3-I both *in vivo* and *in vitro*. The activating or inhibiting effect of SNT on autophagy depending on the cell type was reported (Hou et al., 2019). In this present study, we found that although the increasing ratio of LC3-II to LC3-I may indicate autophagy activation, paradoxically, the expression of p62 was also elevated both *in vivo* and *in vitro* following SNT treatment, which is frequently negatively correlated with the expression of LC3-II. These results indicated that the autophagic flux may be inhibited at the late stage, involving SNT-induced cardiomyocyte cytotoxicity. In addition, mCherry-GFP-LC3 double-labeled adenovirus assay demonstrated that SNT could trigger a dramatic increase of autophagosomes and without alteration of autolysosomes in H9c2 cardiomyocytes. Thus, the increasing ratio of LC3-II to LC3-I in this study may indicate a blockade of autophagic flux by SNT, where fusion of autophagosome with the lysosome may be impeded and cytoplasmic autophagosomes will accumulate. However, the decreased ratio of LC3-II to LC3-I may suggest a fluent autophagic flux in this situation. In fact, previous studies have suggested an association between heart injury and inhibition of late-stage autophagic flux (Jacob et al., 2016; Thakur et al., 2018; Yang et al., 2019a). Taken together, we concluded that SNT induced late-stage inhibition of autophagic flux involving cardiomyocytes in its cytotoxicity.

Recently, EMPA has been shown to regulate autophagy in cardiac tissue and cardiomyocytes (Jiang et al., 2021), which prompts us to make the hypothesis that EMPA may exert potentially cardioprotective effects against SNT *via* improving autophagic flux in cardiomyocytes. As shown in the results, EMPA significantly restored SNT-induced LC3-II to LC3-I ratio elevation and the autophagosome accumulation in cardiomyocytes, indicating more fluent autophagic flux. It is well known that AMPK is a central arbitrator of the cellular response to nutrient availability, and the AMPK–mTOR signaling pathway is one of the classic core molecular regulators of autophagy (Kerkela et al., 2009; Kim et al., 2011). A previous study has reported that SNT can inhibit AMPK activation (Laderoute et al., 2010) and that excessive activation of AMPK could ameliorate SNT-induced cardiomyocyte cytotoxicity (Kerkela et al., 2009). To further explore the underlying mechanism that mediated EMPA’s regulation of SNT-induced autophagic dysfunction, we examined the expression of the AMPK–mTOR autophagy pathway. EMPA was found to restore the inhibition of AMPK and activation of mTOR induced by SNT, while AMPK or autophagy inhibitors blocked this restoration of EMPA. Therefore, the relationship between the EMPA’s anti-cardiotoxic effect against SNT and AMPK-mTOR signaling–mediated autophagy was strongly supported by this *in vitro* study. However, these effects should be further validated *in vivo*.

Although EMPA was shown to exert encouraging cardioprotective effects on SNT-induced cardiotoxicity in this present study, one question still remains unsolved that whether co-treatment with EMPA would affect the anticancer efficacy of SNT. A recent meta-analysis of randomized controlled trials demonstrated no significantly detrimental effect from SGLT2 inhibitors on the incidence of malignancies in general, or in bladder cancer in particular (Dicembrini et al., 2019). Furthermore, EMPA was shown to inhibit the migration and induce the apoptosis of cervical carcinoma cells (Xie et al., 2020). However, further investigations are still required to validate EMPA as a safe drug for patients receiving SNT and to exclude the possible weakening effects of EMPA on SNT’s tumor-killing potency.

## Conclusion

In conclusion, this present study demonstrated that SNT could induce high blood pressure and left ventricular dysfunction in mice and cell viability loss in H9c2 cardiomyocytes *via* inhibition of autophagic flux, whereas EMPA was shown to alleviate the negative effects caused by SNT *via* regulating AMPK–mTOR signaling pathway–mediated cardiomyocyte autophagy.

## Data Availability

The original contributions presented in the study are included in the article/Supplementary Material; further inquiries can be directed to the corresponding authors.

## Abbreviations

SGLT2, sodium–glucose cotransporter-2; EMPA, empagliflozin; SNT, sunitinib; LVF, left ventricular function; SBP, systolic blood pressure; DBP, diastolic blood pressure; MBP, mean blood pressure; HR, heart rate; LV, left ventricular; LVEF, LV ejection fraction; FS, fractional shortening; IVSd, interventricular septal thickness in diastole; IVSs, interventricular septal thickness in systole; LVIDd, LV internal diameter in diastole; LVIDs, LV internal diameter in systole; LVPWd, LV posterior wall thickness in diastole; LVPWs, LV wall thickness in systole; PWD, pulsed wave Doppler; MV E/A ratio, the ratio of blood flow velocity through the mitral valve during early (E) vs. late (A) diastole; DT, deceleration time; IVRT, isovolumetric relaxation time; CFR, coronary flow reserve; cTnT, cardiac troponin T; NT–pro-BNP, N-terminal pro–B-type natriuretic peptide; CC, compound C; Baf A1, bafilomycin A1.
