# Supplementary figures and images for: Sodium–Glucose CoTransporter-2 Inhibitor Empagliflozin Ameliorates Sunitinib-Induced Cardiac Dysfunction via Regulation of AMPK–mTOR Signaling Pathway–Mediated Autophagy
Source: Front Pharmacol. 2021 Apr 29;12:664181. doi: 10.3389/fphar.2021.664181 (PMC8116890; doi:10.3389/fphar.2021.664181)

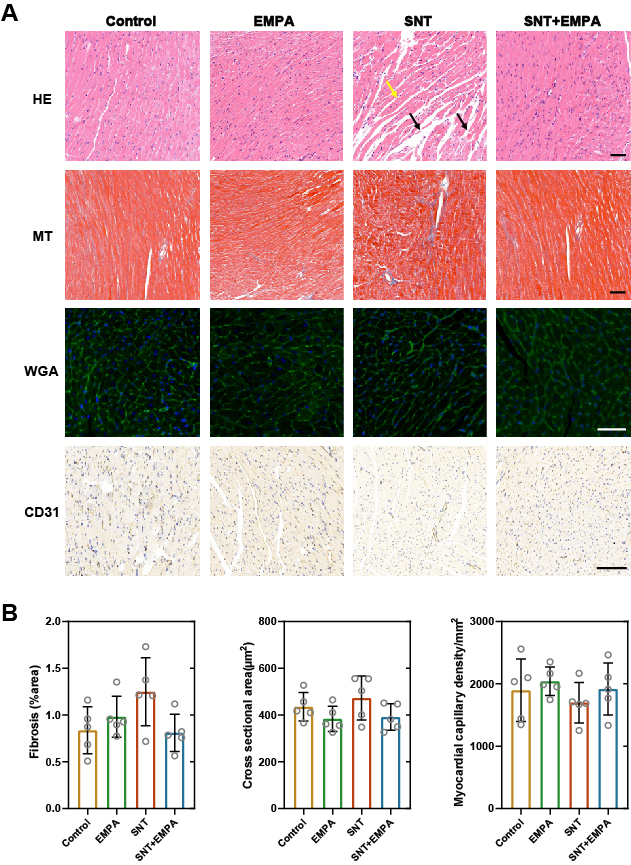

Supplement: Supplementary file 1 [file Image3.JPEG]

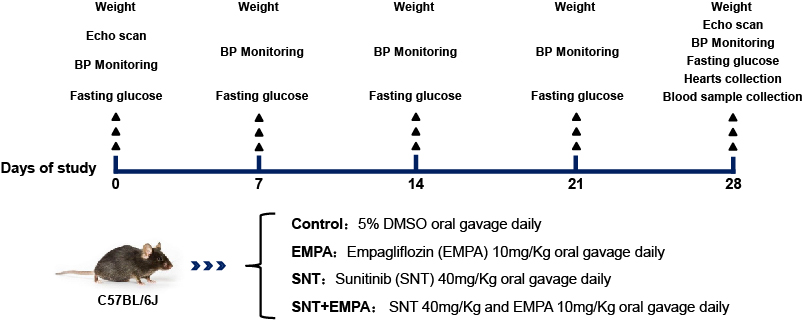

Supplement: Supplementary file 2 [file Image1.JPEG]

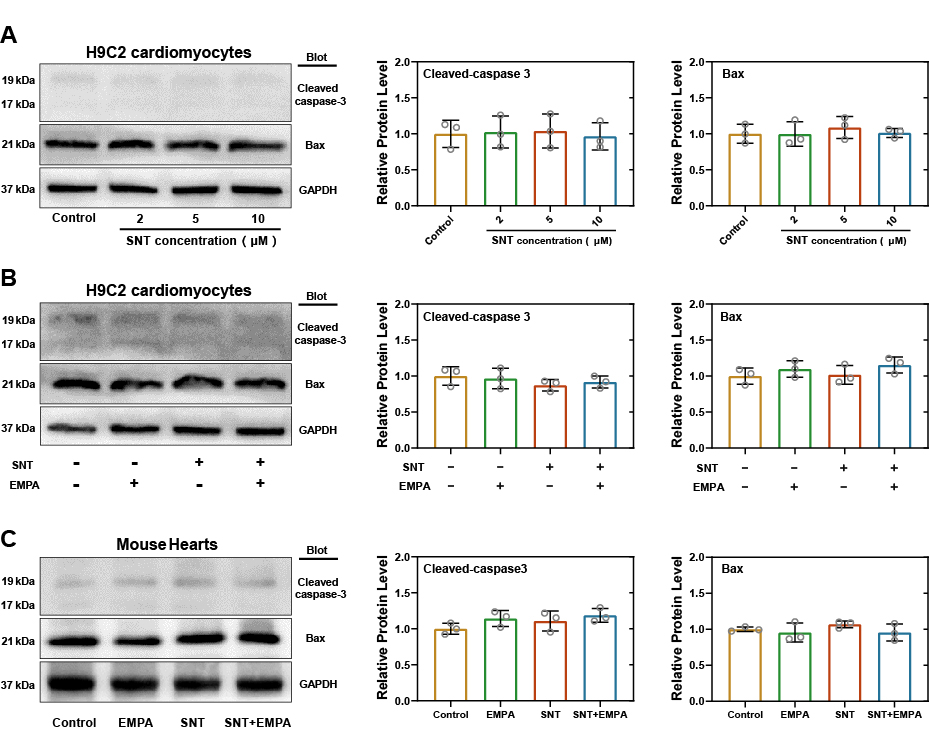

Supplement: Supplementary file 3 [file Image4.JPEG]

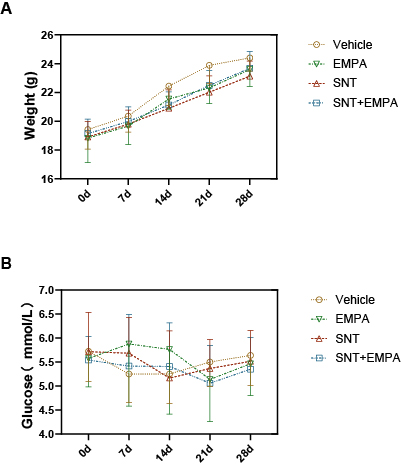

Supplement: Supplementary file 4 [file Image2.JPEG]

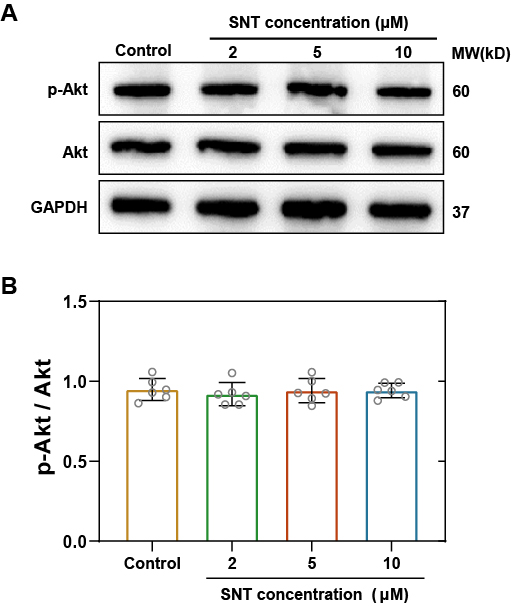

Supplement: Supplementary file 5 [file Image5.JPEG]
