# Supplementary material for: Sodium–Glucose CoTransporter-2 Inhibitor Empagliflozin Ameliorates Sunitinib-Induced Cardiac Dysfunction via Regulation of AMPK–mTOR Signaling Pathway–Mediated Autophagy
Source: Front Pharmacol. 2021 Apr 29;12:664181. doi: 10.3389/fphar.2021.664181 (PMC8116890; doi:10.3389/fphar.2021.664181)
Supplement: Supplementary file 6 [file DataSheet1.docx]

**Supplementary information**

**Additional file :**

**Figure legends**

**Figure S1. Study protocol.**

The experimental studying design of EMPA’s cardioprotective effects on SNT-induced cardiac dysfunction in mice.

**Figure S2. Changes of body weight and blood glucose.**

(A) The body weight curve at different study time points among different groups. n=8 or 12 per group; (B) The blood glucose curve at different study time points among different groups. n=8 or 12 per group.

**Figure S3. Histological sections.**

(A) Representative images of Hematoxylin / Eosin (HE) staining, Masson’s trichrome (MT) staining , Wheat Germ Agglutinin (WGA) staining and representative immunohistochemistry of CD31, bar=50 μm. Yellow arrow, cardiomyocytes showed deepened cytoplasmic staining. Black arrow, cardiomyocytes showed punctate necrosis. (B) Statistical analysis of myocardial fibrosis, cardiomyocyte size and microvascular density among different groups. n=5 per group.

**Figure S4. Protein expression of apoptotic markers.**

(A) Representative western blots of apoptotic markers in H9c2 cardiomyocytes treated with different concentrations of SNT and (B) statistical analysis. n=3 per group; (C) Representative western blots of apoptotic markers in H9c2 cardiomyocytes treated with vehicle, EMPA ,SNT or SNT plus EMPA and (D) statistical analysis. n=3 per group; (E) Representative western blots of apoptotic markers in mice hearts treated by vehicle, EMPA, SNT or SNT plus EMPA and (F) statistical analysis, n=3 per group.

**Figure S5. Protein expression of Akt and phosphorylated Akt in H9c2 cardiomyocytes.**

(A) Representative western blots of Akt and phosphorylated Akt in H9c2 cardiomyocytes treated with different concentrations of SNT; (B) Protein level quantifications of phosphorylated Akt to total Akt ratio, n=6 per group.
